# Mapping the Finer-Scale Carcinogenic Risk of Polycyclic Aromatic Hydrocarbons (PAHs) in Urban Soil—A Case Study of Shenzhen City, China

**DOI:** 10.3390/ijerph17186735

**Published:** 2020-09-16

**Authors:** Dongxiang Chen, Han Zhao, Jun Zhao, Zhenci Xu, Shaohua Wu

**Affiliations:** 1 Key Laboratory of Urban Land Resources Monitoring and Simulation, Ministry of Land and Resources, Shenzhen 510034, China; 20190302@zufedfc.edu.cn (D.C.); njuzhaohan@126.com (H.Z.); eversuncool@hotmail.com (J.Z.); 2 School of Business Administration, Zhejiang University of Finance & Economics Dongfang College, Haining 314408, China; 3 Department of Geography, University of Hong Kong, Hong Kong 999077, China; xuzhencinl@gmail.com; 4 Institute of Land and Urban-rural Development, Zhejiang University of Finance & Economics, Hangzhou 310018, China

**Keywords:** risk assessment, spatial analysis, PAHs, carcinogenic risk

## Abstract

The high-precision mapping of urban health risk is a difficult problem due to the high heterogeneity of the urban environment. In this paper, the spatial distribution characteristics of the Polycyclic Aromatic Hydrocarbon (PAH) content in the urban soil of Shenzhen City were analyzed through a field investigation. We propose an approach for improving the accuracy and spatial resolution of PAH carcinogenic risk assessment by integrating the pollutant distribution and Location Based Service (LBS) data. The results showed that the concentration of PAHs in the high-density urban area was 271.67 ng g^−1^, which was 27.2% higher than that in the green area. Although the average carcinogenic risk of PAHs in the surface soil of Shenzhen city was less than 10^−6^, the maximum carcinogenic risk at some sample sites exceeded 10^−6^, which indicates a potential health risk. The LBS data were effective for high-precision mapping of the population distribution. According to the combination relationship between the risk threshold of pollutants and the population density, four types of risk zones were proposed. Among them, 6.9% of the areas had a high-risk and high population density and 15.8% of the areas were high-risk with a low population density. These two kinds of zones were the critical areas for controlling risk. The fine-scale risk mapping approach for determining the carcinogenic risk of soil PAHs integrating pollutant distribution and location based service data was demonstrated to be a useful tool for explicit spatial risk management. This tool could provide spatial insights and decision support for urban health-risk management and pollution prevention.

## 1. Introduction

Polycyclic Aromatic Hydrocarbons (PAHs) are prevalent in the urban environment and have become one of the main pollutants in cities [1,2]. PAHs are a popular research topic due to their carcinogenic, teratogenic and mutagenic toxicity to humans [3]. Soil provides an important pool of PAHs and accounts for 90% of the PAHs in the terrestrial environment [4]. Usually, concentrations of PAHs in urban soil are higher than those in other soils because urban environments emit more PAHs from industry, transportation and human activities. The PAHs in urban soil can affect human heath through the water, atmosphere, food and other media [5]. Therefore, the PAHs in urban soil represent a risk to the health of the urban population. Mapping the finer-scale carcinogenic risk of PAHs could thus provide critical information for local environmental protection and public health management [6,7,8].

A number of studies have shown that the main sources of PAHs are the largescale emissions of man-made pollution [9,10]. As the main gathering places of human beings, urban areas suffer PAH pollution that is continuously aggravated by high-intensity industrial activities, heavy traffic and human activities [11,12]. Many scholars have found that the PAH concentration in urban soil is much higher than that in the background soil [11,12,13]. A number of studies have explored the distribution, sources and risk assessment of PAH pollution in urban soil, indicating that the PAHs in urban soil are deeply affected by human activities and are often concentrated near industrial zones and highways [14,15,16]. Previous studies have deepened our understanding of the risk perception of urban PAH pollution [17] and increasingly more attention is being given to the carcinogenic risks caused by urban organic pollution [18]. To better identify and manage the risk of urban soil pollution, high-precision mapping is required. However, previous risk assessments mainly focused on risk probability and lacked consideration of the spatial distribution information of the population. It was thus difficult to determine the fine-scale carcinogenic risk of the urban soil pollution. However, health-risk mapping needs accurate information on pollutant and population distributions. Moreover, it is difficult to obtain population data with a high spatial resolution. As a result, previous health-risk mapping considering the spatial distribution of pollution lacked the presence of a well-thought-out population distribution. Now with the application of big data, it is possible to accurately obtain the spatial distribution of people [19,20,21]. Location Based Service (LBS), for example, can indicate the exact population distribution [22,23]. This spatially refined mapping of the population distribution provides an important entry point. Therefore, we propose a technical method for mapping the high spatial resolution carcinogenic risk of PAHs in urban areas by combining population density mapping with LBS and the spatial analysis of PAHs.

High-resolution health-risk mapping is a useful tool for the urban environment and public health management. This paper uses Shenzhen, a typical city that has been rapidly urbanized in China, as an example. We carried out a soil field survey and combined LBS data with the pollutant distribution to achieve high spatial resolution health-risk mapping. The main objectives were (1) to analyze the spatial distribution of PAHs, (2) to draw a high resolution spatial map of the population using LBS data and (3) to propose technology for integrating the pollution distribution with LBS information for carcinogenic risk mapping.

## 2. Materials and Methods

### 2.1. Collection and Preparation of the Urban Soil Samples

**Figure 1:**
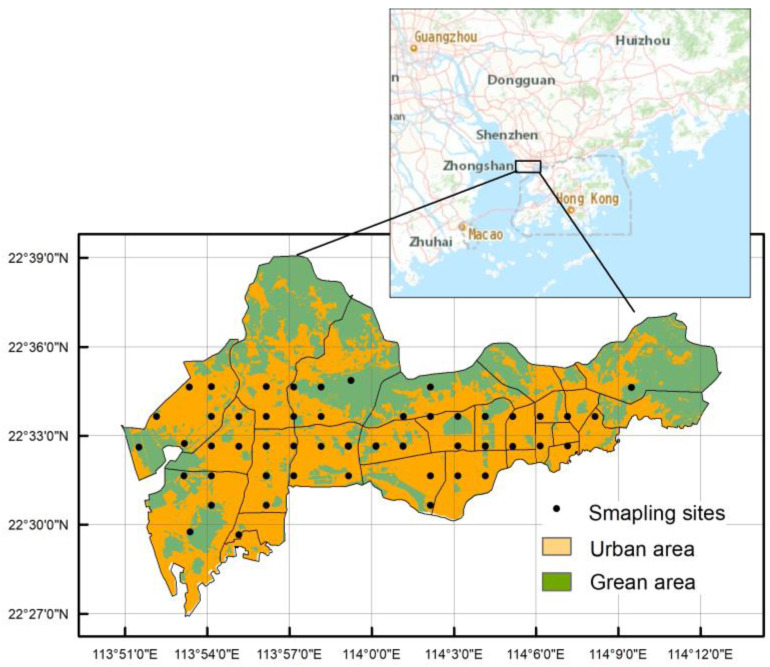
Research area and sampling sites.

Shenzhen (N 22°32′, E114°03′) is located in the southern portion of Guangdong Province, China, on the eastern shore of the Pearl River Delta (Figure 1). Based on the field investigations, 61 surface soil samples (0–5 cm) were collected in the main urban area of Shenzhen (Luohu, Futian, Nanshan and Baoan) from different land use types. The sampling sites uniformly covered the study area. Among them, 46 samples were taken from a high-density urban area (residential land, industrial land, commercial land and traffic land) and 15 samples were taken from green area land. In the sampling process, GPS was used to locate the sampling sites. The distribution of the sampling sites is shown in Figure 1.

The five-point sampling method was adopted for sampling. Each sample site was divided into a square plot of a certain size and samples were taken at the center and four corners of the square and thoroughly mixed. A mixed sample of 1 kg was collected as the sample to be tested. The soil samples were naturally air-dried in the shade of the room. The samples were ground and screened using 20, 60 and 100 meshes. The samples were placed in brown glass bottles together with the original samples and stored in an environment of −4 °C as a reserve. The soil samples tested for PAH content used 100 mesh sieves [24].

### 2.2. Chemicals and Materials

Mixed standard samples were purchased for 16 PAHs—naphthalene (Nap), acenaphthylene (Acy), acenaphthene (Ace), fluorene (Fl), phenanthrene (Phe), anthracene (Ant), fluoranthene (Flu), pyrene (Pyr), benz[a]anthracene (BaA), chrysene (Chr), benzo[b]fluoranthene (BbF), benzo[k]fluoranthene (BkF), benzo[a]pyrene (BaP), indeno[1,2,3-cd]pyrene (InP), dibenz[a,h]anthracene (DBA), and benzo[g,h,i]perylene(BP). The PAHs’ recovery indicators were chosen from five mixed samples—naphthalene-d8, acenaphthene-d10, phenanthrene-d10, chrysene-d12 and perylene-d12. Silica gel (100–200 mesh) was extracted via dichloromethane, n-hexane and acetone before use and then dried in a ventilated kitchen and stored in n-hexane after 18 h of activation in an oven. Anhydrous sodium sulfate was burned in a muffle oven at 450 °C for 5 h and placed in a dryer for use.

### 2.3. Sample Treatment 

The 5 g soil sample was accurately weighed, mixed with 5 g of anhydrous sodium sulfate and supplemented with recovery indicators. A 100 mL mixture of n-hexane and dichloromethane (volume ratio, 1:1) and a 2 g activated copper sheet were successively added and extracted in a cable extractor for 24 h. After the extract was concentrated to 2 mL on a rotary evaporator, 10 mL of n-hexane was added and further concentrated to 1–2 mL to convert the solvent. The concentrated extract was then added to a silica gel chromatography column for separation and purification and was first eluted with 15 mL n-hexane without collection. Then, 50 mL dichloromethane and an n-hexane mixture solution (volume ratio 2:3) were eluted and the eluent was collected. The collected eluent was converted into a solvent with n-hexane, blown to 1 mL with high purity nitrogen, put into a GC bottle and tested on machine [25].

The PAHs in the concentrate were quantitatively analyzed using a Shimadzu QP2010 Ultra. The column was an Rtx-5MS (30 m × 0.5 mm ID × 0.25 ms). The carrier gas was helium and the injection volume was set to 1 micron. The chromatographic column heating program was set as follows—from 60 °C to 200 °C at 5 °C·min^−1^, then to 250 °C at 2 °C·min^−1^ and then to 290 °C at 20 °C·min^−1^ and maintained for 20 min. The temperature of the injection port rose from 100 °C to 280 °C at 200 °C·min^−1^ and was maintained for 40 min.

In the sample pretreatment process, blank and matrix blank experiments were performed every 6 samples. We repeated this process every 12 samples and retested if the deviation exceeded 15%. The recovery rates for the five recovery indicators in all samples were as follows—naphthalene-d8, 50% ± 13%; Acenaphthene-d10, 58% ± 19%; Phenanthrene, d10 71% ± 21%; Chrysene, d12 75% ± 18%; and Perylene, d12 93% ± 27%.

### 2.4. Carcinogenic Risk Assessment

The incremental lifetime cancer risk (ILCRs) model is widely used to assess the carcinogenic risk of PAHs in an environmental media [26,27]. Ingestion (ILC_Ringestion_), dermal exposure (ILCR_dermal_) and inhalation (ILC*Rinhalation*) are the main pathways of pollutant exposure. The calculation equations for each exposure pathway are as follows [28]:

(1) $$ILCR_{ingestion}\;=\;\frac{CS\times CSF_{ingestion}\times \sqrt[{3}]{ABW/70}\times IR_{ingestion}\times EF\times ED}{ABW\times AT\times 10^{6}}$$

(2) $$ILCR_{inhalation}\;=\;\frac{CS\times CSF_{inhalation}\times \sqrt[{3}]{ABW/70}\times IR_{inhalation}\times EF\times ED}{ABW\times AT\times PEF}$$

(3) $$ILCR_{dermal}\;=\;\frac{CS\times CSF_{dermal}\times \sqrt[{3}]{ABW/70}\times SA\times AF\times ABS\times EF\times ED}{ABW\times AT\times 10^{6}}$$

(4) $$\mathrm{CS}\;=\sum \left({\mathrm{PAH}}_{i}\times {\mathrm{TEF}}_{i}\right)$$

(5) $$\mathrm{CR}\;=\sum ({\mathrm{ILCR}}_{ingestion}+{\mathrm{ILCR}}_{inhalation}+{\mathrm{ILCR}}_{dermal}),\;$$

where *CS* is the toxic equivalent concentration of PAH in the soil (ugg^−1^); TEF is the toxic equivalent coefficient of the PAH monomer equivalent to BaP (shown in Table 1); and *CSF_ingestion_*, *CSF_inhalation_*_and_ *CSF_dermal_* are the carcinogenic slope factors (mg·kg^−1^·day^−1^). ABW is the average body weight (kg), IRingestion is the daily ingestion rate (mg·day^−1^) and IRinhalation is the daily inhalation rate (m^3^·day^−1^). *EF* is the exposure frequency (day·year^−1^), *ED* is exposure duration (year), *AT* is the life expectancy (day), PEF is the production factor of soil dust (m^3^·kg^−1^), *SA* is the dermal surface area (cm^2^·day^−1^), *AF* is the soil adhesion factor (mg·cm^−2^) and ABS is the dermal absorption coefficient. The specific values of each parameter for children and adults are detailed in Table 2. The values of these parameters were obtained from previous research [29,30]. According to the US Environmental Protection Agency, based on a one in a million chance of additional human cancer over a 70-year lifetime, the carcinogenic risk (CR) classification criteria of CR are as follows—CR < 10^−6^ indicates that the health risk is acceptable [31].

### 2.5. Finer-Scale Population Density Mapping and Carcinogenic Risk Mangemet Zoning

According to the statistical report on the development of China’s Internet report, by December 2018, China’s mobile Internet users had reached 817 million. Further, more than 71% of people over the age of 16 have mobile phones [32]. The proportion in urban areas was noted to be higher than that in rural areas. Therefore, the spatial distribution of urban population can be accurately evaluated by using mobile location data. The distribution map of the adult population is obtained through the location-based service (LBS) platform (Baidu, Beijing, China). Baidu provides a population density distribution index (PDI) to characterize the spatial distribution patterns of mobile phone users. However, this is not real population density data. Thus, we need to convert the population distribution index into population density. Statistical departments possess the adult population statistics of cities. Based on the population index, the statistical population can be allocated to spatial distribution. The formula is as follows:

(6) $$Pop.Dens\left(x,y\right)\;=\;Stati.Pop\times \frac{PDI\left(x,y\right)}{\sum PDI\left(x,y\right)},$$

where Pop.Dens(x,y) is the distribution of the adult population at location(*x*,*y*), Stati.Pop is the number of adults in the population from the statistical yearbook and LBS(x,y) is the PDI value of a point location *(x,y)*. ∑LBS(x,y) is the sum of the PDI value in the research area.

Due to the lack of accurate population spatial distribution data, the impact of population density on risk was seldom considered in previous health risk assessments. In fact, the greater the population density is, the greater the health risk of the exposed population. Therefore, we introduced population density into the process of risk assessment to determine the risk of spatial distribution. By multiplying the carcinogenic risk (*CR*) by the population density, we can obtain the health risk density (HRD), which indicates the number of people who may have carcinogenic risks in the unit area:

(7) Health risk density(x,y) = Pop.Dens(x,y)×CR(x,y)

In addition, a simple spatially explicit zoning approach is provided for urban soil health-risk management based on the *CR* index and population density (Table 3). According to the threshold value of the health risk index (10^−6^) and population density (200 people ha^−1^), four regions are delineated—high-risk high population density, high-risk low population density, low-risk high population density and low-risk low population density. The spatially explicit management zones integrating carcinogenic PAH risk and population density provide a useful tool for the spatially explicit management of public risk. 

## 3. Results and Discussion

### 3.1. Statistical Characteristics of PAHs

Soil samples were divided into two groups—high-density urban areas and green areas. The Statistical characteristics of PAHs are shown in Table 4. In the urban area, the 16USEPA priority PAHs (Σ16PAHs) varied widely from 92.84 to 2309.88 ng·g^−1^, with a mean of 526.32 ng·g^−1^. Carcinogenic PAHs (PAHscarc: BaA, Chr, BbF, BkF, BaP, InP and DBA) ranged from 24.45 to 1274.96 ng·g^−1^, with a mean value of 271.67 ng·g^−1^. PAHscarc accounted for 51.6% of the total PAHs. The PAH compounds were divided into five groups according to the number of their aromatic rings. Carcinogenic PAHs are 4-ring or 5-ring PAHs. In terms of the relative composition of the soil, 4- and 5-ring PAHs dominated with 39.5% and 26.6%, respectively, followed by 6- and 3-ring PAHs at 16.4% and 12.9%, respectively. Two-ring PAHs were the least prevalent and only accounted for 4.6%.

In the green area, the Σ16 PAHs ranged from 73.47 to 985.05 ng g^−1^, with a mean of 396.27 ng·g^−1^. The carcinogenic PAH PAHscarc varied from 24.7 to 616.27 ng g^−1^, with a mean value of 213.45 ng. carcinogenic PAHs accounted for 51.6% of the total PAHs. In terms of the relative compositions of the soils, 4- and 5-ring PAHs accounted for 34.9% and 28.3%, respectively, followed by 6- and 3-ring PAHs at 19.1% and 13.0%, respectively. The 2-ring PAHs only accounted for 4.7%. 

Comparing the urban area and green areas, significant differences in polycyclic aromatic hydrocarbon concentrations were found in the two regions. The mean of the PAHs in the urban area was 526.32 ng·g^−1^, which was higher by 32.8% than the mean in the green area. The carcinogenic PAH concentration in the high-density urban areas was 271.67 ng·g^−1^, which was 27.2% higher than that in the green area. This shows that in urban areas, both the total amount and the amount of carcinogenic PAHs were significantly higher than those in the green space. The high-density urban area was the area with both a higher population density and a higher PAH concentration. The Identifying the relationship between health risk and population density would provide insights for health-risk management in urban areas.

### 3.2. Spatial Distribution of PHAs

**Figure 2:**
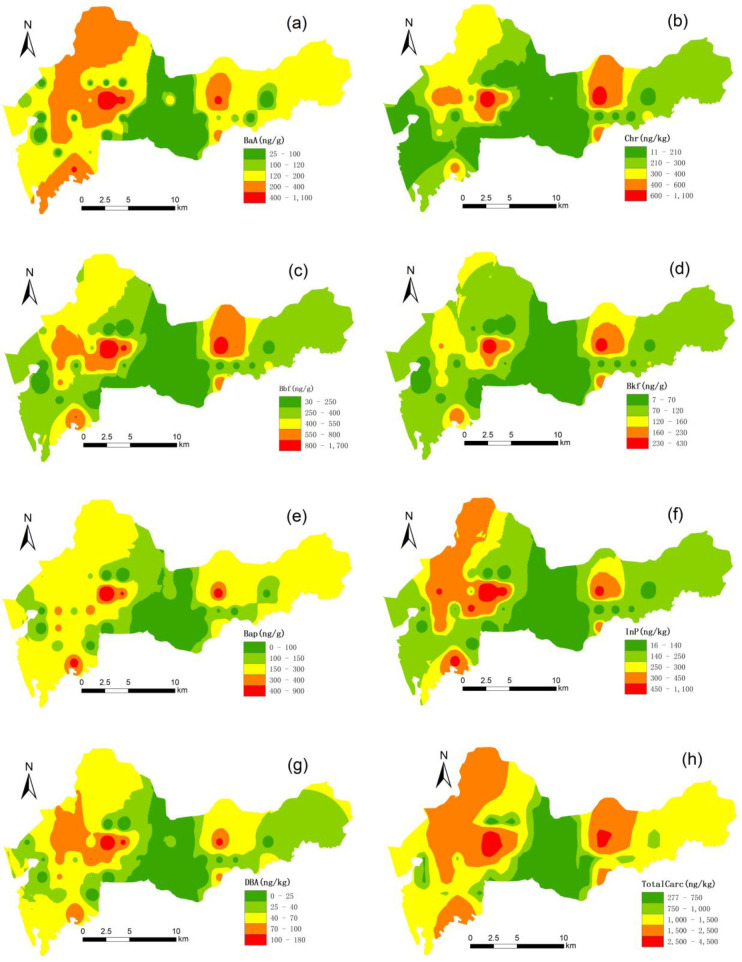
Spatial distribution of carcinogenic PAH concentrations (benz[a]anthracene, BaA (**a**); chrysene, Chr (**b**); benzo[b]fluoranthene, BbF (**c**); benzo[k]fluoranthene, BkF (**d**); benzo[a]pyrene, BaP (**e**); indeno[1,2,3-cd]pyrene, InP (**f**); dibenz[a,h]anthracene, DBA (**g**) and total carcinogenic PAHs (**h**)).

We compared four spatial interpolation methods (Inverse Distance Weight, Local polynomial, Sample kriging and Kernel Smoothing) to predict the spatial distribution of the PAHs and found that Inverse Distance Weight had the lowest prediction errors. Inverse Distance Weight is a mathematical function that assumes closer values are more closely related than further values. Inverse Distance Weight is widely used in the spatial analysis of pollution distribution [33]. The spatial distributions of seven carcinogenic PAHs (BaA, Chr, BbF, BkF, BaP, InP and DBA) and PAHs (Σ7cPAHs) were determined by the Inverse Distance Weight method shown in Figure 2. The PAHs had great spatial variability. Based on the spatial distribution patterns, the PAH concentrations in the northeast and northwest of the research area were high, while the concentrations in the central region were low. The spatial distributions of high value areas and low value areas were relatively similar. This distribution pattern was controlled by the process of urbanization, land use and the properties of the PAHs. Carcinogenic PAHs are 4-ring or 5-ring PAHs and have similar properties, so there were also some similarities in their spatial distribution patterns. The concentration of polycyclic aromatic hydrocarbons (PAHs) was higher in industrial and traffic-intensive areas but lower in areas with large green space, indicating that the land-use differences in rapid urban processes have a significant impact on PAH accumulation.

### 3.3. Carcinogenic Risk Assessment

According to the exposure assessment, the carcinogenic risks of ingestion, inhalation and dermal exposure were calculated. The carcinogenic risk and total risk of PAHs in adults based on three different exposure pathways is shown in Table 5. Among the three different exposure routes, the skin exposure route had the highest carcinogenic risk, accounting for 64.0% of the total risk. Ingestion exposure was the second, accounting for 36.0% of the total risk, while the carcinogenic risk of the inhalation exposure pathway was much lower than that of the other two exposure pathways, meaning that this pathway can be ignored. According to the proportion of exposure pathways, we suggest avoiding direct dermal contact with the soil or ingesting soil dust. The average risk of PAHs in the topsoil of Shenzhen city was less than 10^−6^. However, the maximum values of local points for adult carcinogenic risk exceeded the risk threshold, indicating that there were potential carcinogenic risks in some areas. Therefore, we need to further identify where the risks exceeded the risk threshold of PAHs and how many people are currently exposed to such carcinogenic risks.

### 3.4. Mapping the Finer-Scale Carcinogenic Risk of PAHs

The carcinogenic risk assessment can represent the overall situation but it is difficult to express how many people are exposed to such risk in spatial detail. Risk management is more important in areas where the pollutants exceed the risk threshold and the population density is higher. Therefore, identifying the critical areas for health-risk management not only relates to the concentration of pollutants but must also consider the distribution density of the population. In the past, the impact of population distribution on health risk was often ignored in health risk assessments, mainly due to the lack of high-resolution population distribution data. With the growing popularity of mobile phones, it has become possible to use LBS data to determine population density with a fine spatial resolution.

**Figure 3:**
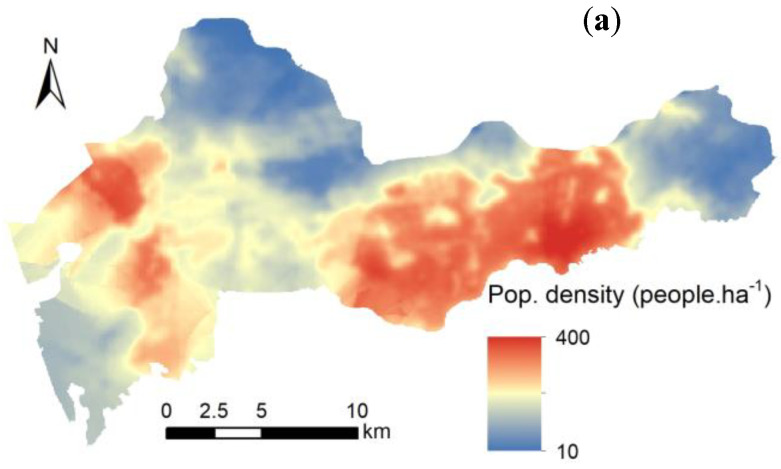

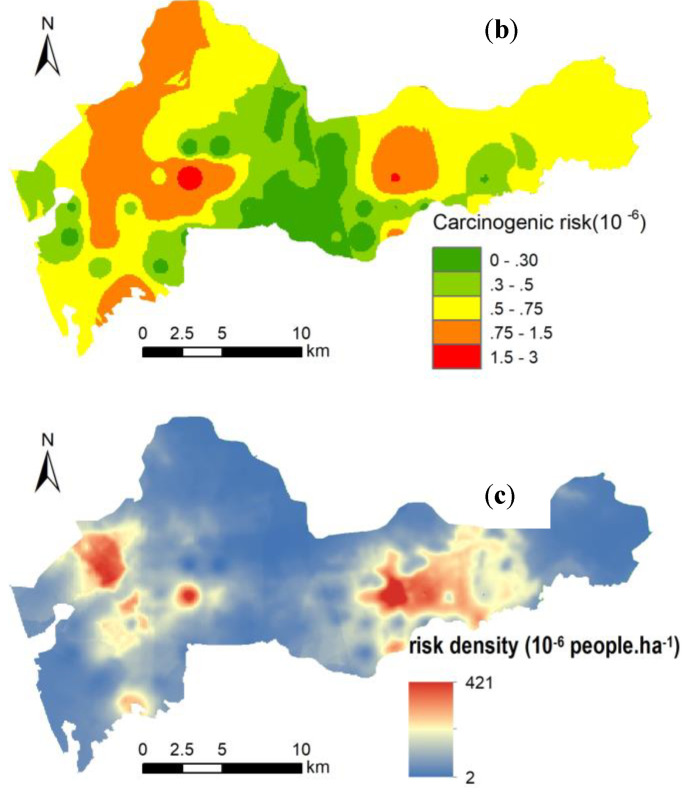
Maps of Location Based Service (LBS) and PAH risk distribution (**a**) Population density obtained from LBS, (**b**) Carcinogenic risk of PAHs and (**c**) Health risk density (HRD).

Traditional demographic data are often based on administrative unit surveys, which do not accurately represent the fine-scale distribution of the population. We used the LBS data provided by Baidu (http://lbsyun.baidu.com) and official population statistics data to establish a relationship equation between the LBS and statistical data and then drew a high-resolution population distribution map (shown in Figure 3b). The accuracy of the LBS data was greater than 80% (shown in Figure 3a). Multiplying the CR (Figure 3c) and population density (Figure 3b), we further calculated the health risk density (HRD), which represents the number of people at risk of cancer per unit area (Figure 3c). This HRD map can more intuitively express the spatial population distribution patterns of PAH carcinogenic risk.

**Figure 4:**
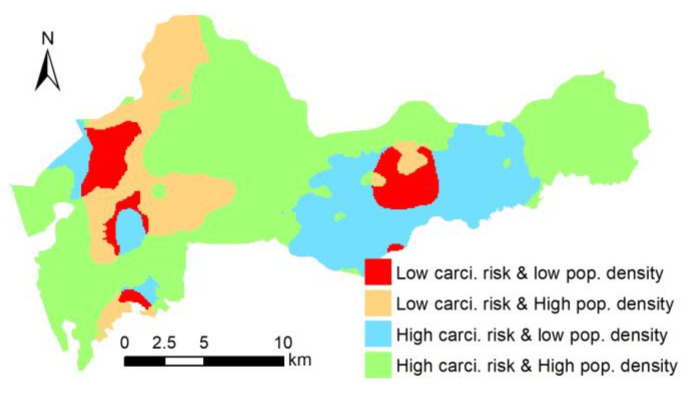
Spatially explicit management zones integrating carcinogenic risk and population density.

In addition, to facilitate better management according to the combination relationship between the health risk index and population density, we divided the area into four types of risk management zones—high risk with high population density, high risk with low population density, low risk with high population density and low risk with high population density (shown in Figure 4). The high risk–high population density group accounted for 6.9% and was the most critical area of risk management. Because of the high population density in these areas, the number of people at risk of cancer is also high [13,34]. Further, 15.8% of the area was high risk–low population density. This zone also needs attention to remind the public to reduce their exposure to soil. The low risk–high population density and low risk–high population density zones accounted for 21.4% and 55.8%, respectively. These two areas are safe but their continuous accumulation of pollutants should be controlled. Risk management zones integrating carcinogenic PAH risk and population density provide a useful tool for the spatially explicit management of public risk.

## 4. Uncertainty and Prospects

Although the approach combining pollutant distribution and LBS data can effectively characterize the risk of health exposure to PAHs, the uncertainty in this study requires attention. Since mobile phones are mainly owned by adults, the LBS data predominantly reflect the distribution of the adult population. It is thus difficult to characterize the distribution of children. Indeed, children are the most vulnerable group for health risks because they are exposed to the soil environment frequency and the tolerated dose of PAHs for children is small [35]. In addition, there is also uncertainty in the use of the exposure parameters, which were mainly obtained from the literature data due to a lack of local parameters. These parameters could affect the accuracy of carcinogenic risk evaluation. At present, China lacks the parameters for localized health risk evaluations and it is recommended that China carry out special research to form a set of local parameters to evaluate the carcinogenic risk of polycyclic aromatic hydrocarbons and thus improve the fine-management level of health risks [35]. Considering the lack of data on children and other issues, children’s population distribution and pollution exposure can be inferred by methods such as big data and machine learning, including the kindergarten distribution, family composition and commodity purchases. In short, despite the uncertainty of current research, LBS data is an effective technology for health risk assessment and can provide useful support for the fine-resolution mapping of health-risk management.

Shenzhen is a young city presenting only the short-term accumulation of soil pollution. Moreover, the concentration of soil PAHs is relatively low in the Pearl River Delta urban agglomeration [26]. Cities in other parts of China, such as those on the Bohai Sea Rim and in the Yangtze River Delta, have higher levels of PAHs, which pose a greater risk to human health [35,36]. Presently, the LBSs of large cities are being widely used in the fields of commercial services and urban planning but there are few studies on the application of LBS data to environmental pollution management. In the future, the use of LBS data for the research and management of population health risks will have important application prospects.

## 5. Conclusions

This study collected and tested the concentrations of PAHs in urban areas. Although the variation in different kinds of PAHs is very large, their spatial patterns are closely related to human activities. The concentration levels of PAHs in the industrial and traffic-intensive areas were higher than those in the green space area, indicating that the differences in urban land use have a significant impact on the accumulation of PAHs.

An integrated approach for mapping the finer-scale carcinogenic risk of (PAHs) was proposed by combining the pollutant distribution with LBS data. The method proposed in this study is a useful tool that could provide spatial detail for and insight into, soil pollution investigations and health-risk management. LBS provides effective data for health risks by accurately representing the distribution of the population. Our research showed that the health risks matched the distribution of population density—6.9% of the area had a high population density and high carcinogenic risk and 55.8% of the area had a low health risk and population density. Therefore, sufficient attention should be paid to the critical risk areas in high population density areas.

A shortcoming of our method is that it remains difficult to distinguish between different age group populations using LBS data. Old individuals, middle-aged individuals and children react very differently when exposed to PAHs. Thus, in the future, we will combine multi-source data, such as kindergarten distribution data and population age surveys, to improve the accuracy of health-risk mapping.

## Figures and Tables

**Table 1:** Toxicity equivalent coefficient of carcinogenic Polycyclic Aromatic Hydrocarbons (PAHs).

PAH	BaA	Chr	BbF	BkF	BaP	InP	DBA
TEF value	0.1	0.01	0.1	0.1	1	0.1	1

**Table 2:** Parameters used in the incremental lifetime carcinogenic risk assessment.

Parameters	Unit	Value
average bodyweight (ABW)	kg	58.55
exposure frequency (EF)	day year^−1^	365
Exposure duration(ED)	year	24
daily inhalation rate (IR _inhalation_)	m^3^ day^−1^	13.04
daily ingestion rate(IR _ingestion_)	mg day^−1^	100
dermal surface area(SA)	cm^2^ day^−1^	5700
soil adhesion factor(AF)	mg cm^−2^	0.07
dermal absorption coefficient(ABS)		0.13
life expectancy(AT)	day	80 × 365
production factor of soil dust(PEF)	m^3^ kg^−1^	1.36 × 10^9^
carcinogenic slope factor of ingestion(CSF _ingestion_)	(mg kg^−1^ day^−1^)^−1^	7.3
carcinogenic slope factor inhalation(CSF _inhalation_)	(mg kg^−1^ day^−1^)^−1^	3.85
carcinogenic slope factor dermal exposures (CSF _dermal_)	(mg kg^−1^ day^−1^)^−1^	25

**Table 3:** Risk zoning matrix combining risk threshold and population density.

Order	Risk Threshold	Population Density (People ha^−1^)	Risk Management Zones
1	>10−6	>200	High risk with high pop.Dens
2	>10−6	<200	High risk with low pop.Dens
3	<10−6	>200	low risk with high pop.Dens
4	<10−6	<200	low risk with low pop.Dens

Notes: pop.Dens denotes population density.

**Table 4:** The Statistical characteristics of PAHs.

High-Density Urban (ng g^−1^)					Green Area (ng g^−1^)			
PAHs	Min	Max	Mean	SD	Min	Max	Mean	SD
Nap	11.16	57.29	24.07	9.78	6.37	35.62	18.82	7.80
Acy	0.69	35.17	5.96	6.50	0.89	7.78	3.81	2.45
Ace	ND	7.70	1.68	1.37	0.50	3.71	1.36	0.95
Fl	1.92	17.97	6.86	3.06	2.78	9.79	5.40	2.06
Phe	13.64	184.79	47.25	31.20	12.78	75.32	36.43	17.30
Ant	1.28	27.13	6.39	5.60	1.20	8.68	4.41	2.65
Flu	10.79	355.82	65.09	68.33	9.59	82.19	38.70	22.75
Pyr	8.69	345.27	54.45	59.76	7.88	81.00	34.85	21.30
BaA	3.30	217.21	33.99	37.40	3.96	63.58	24.35	17.29
Chr	3.30	217.78	54.28	49.25	2.24	97.77	40.23	28.01
BbF	5.91	336.82	76.12	73.35	5.68	184.93	60.56	49.17
BkF	1.28	85.50	20.17	19.42	1.21	38.94	14.83	10.60
BaP	2.45	171.87	34.22	33.58	ND	94.62	28.97	24.28
InP	3.47	210.55	43.58	41.48	3.11	119.95	36.67	31.64
DBA	0.25	35.23	9.30	8.22	ND	25.36	7.84	6.92
BP	3.25	169.35	42.90	37.06	1.22	102.71	39.04	31.74
∑PAHs	92.84	2309.88	526.32	457.12	73.47	985.05	396.27	262.44
∑PAHscarc	24.45	1274.96	271.67	255.35	24.70	616.27	213.45	164.43

Notes: SD denotes standard deviation; ND denotes not detected (below the detection limit).

**Table 5:** Incremental lifetime cancer risk (ILCRs) and carcinogenic risk (CR) in different exposure pathways for adults.

Exposure Pathway	Min	Max	Mean
ILCR_dermal_	1.4 × 10^−8^	1.78 × 10^−6^	3.57 × 10^−7^
ILCR_ingestion_	7.9 × 10^−9^	1.1 × 10^−6^	2.01 × 10^−7^
ILCR_inhalation_	4 × 10^−13^	5.08 × 10^−11^	1.02 × 10^−11^
Total exposure (CR)	2.19 × 10^−8^	2.79 × 10^−6^	5.58 × 10^−7^
